# Effects of Saturation Levels on the Ultrasonic Pulse Velocities and Mechanical Properties of Concrete

**DOI:** 10.3390/ma14010152

**Published:** 2020-12-31

**Authors:** Ma. Doreen Esplana Candelaria, Seong-Hoon Kee, Jurng-Jae Yee, Jin-Wook Lee

**Affiliations:** 1 Department of ICT integrated Ocean Smart Cities Engineering, Dong-A University, Busan 49315, Korea; mecandelaria@up.edu.ph (M.D.E.C.); jjyee@dau.ac.kr (J.-J.Y.); 2 Institute of Civil Engineering, University of the Philippines Diliman, Quezon City 1101, Philippines; 3 Principal Researcher, Advanced Railroad Civil Engineering Division, Korea Railroad Research Institute, 176 Cheoldobangmulgwan-ro, Uiwang-si, Gyeonggi-do 16105, Korea

**Keywords:** concrete, saturation level, ultrasonic pulse wave velocity, condition assessment

## Abstract

The main objective of this research is to investigate the effect of water content in concrete on the velocities of ultrasonic waves (P- and S-waves) and mechanical properties (elastic modulus and compressive strength) of concrete. For this study, concrete specimens (100 mm × 200 mm cylinders) were fabricated with three different water-to-binder ratios (0.52, 0.35, and 0.26). These cylinders were then submerged in water to be saturated in different degrees from 25% to 100% with an interval of 25% saturation. Another set of cylinders was also oven-dried to represent the dry condition. The dynamic properties of concrete were then assessed using a measurement of elastic wave accordance with ASTM C597-16 and using resonance tests following ASTM C215-19, before and after immersion in water. The static properties of saturated concrete were also assessed by the uniaxial compressive testing according to ASTM C39/C39M-20 and ASTM C469/C469M-14. It was observed that the saturation level of concrete affected the two ultrasonic wave velocities and the two static mechanical properties of concrete in various ways. The relationship between P-wave velocity and compressive strength of concrete was highly sensitive to saturation condition of concrete. In contrast, S-wave velocity of concrete was closely correlated with compressive strength of concrete, which was much less sensitive to water saturation level compared to P-wave velocity of concrete. Finally, it was noticed that water saturation condition only little affects the relationship between the dynamic and elastic moduli of elasticity of concrete studies in this study.

## 1. Introduction

Concrete, a heterogeneous and porous material, comprises several types of voids (e.g., interface space in C-S-H, capillary voids, entrained air bubbles, and entrapped air voids) [1] that can be penetrated by some other materials such as water. It has been observed that water in concrete pores has an important influence on the mechanical and durability properties of concrete [2]. For instance, more water in concrete could lead to a more susceptible environment that can eventually deteriorate concrete structures. Some deteriorations in concrete are manifested as microcracks and/or enhanced porosity that do not necessarily affect the structural integrity of the whole structural systems. However, those factors could increase the permeability of concrete and allow the material to absorb more water and/or deleterious ingredients, which could accelerate deterioration process from various sources (e.g., carbonation, freezing and thawing, corrosion of reinforcing steels, etc.). Finally, it could induce severe damage in concrete such as horizontal/vertical fracture planes (e.g., delamination defects and surface-breaking cracks) and sectional loss due to concrete spalling, which can substantially degrade its structural performance. Therefore, it is important to monitor health condition of concrete subjected to saturated (partial or full) conditions, and if necessary, to make optimal maintenance actions.

A significant part of the evaluation of the state of conservation and structural performance of these structures is the assessment of their actual mechanical properties (e.g., concrete compressive strength, *f_c_*, and elastic modulus of concrete, *E_c_*) [3]. These properties are usually evaluated by a destructive method (e.g., uniaxial compressive test according to ASTM C39/C39M-20) applied to the extracted core samples from some parts of concrete members. While this method could result in accurate information, it is time-consuming, expensive, and may cause further damage to the structure being monitored. Furthermore, for results to be reliable, it will be necessary to obtain many concrete cores throughout the whole concrete structure, which may substantially increase cost and efforts and limit the use in field practice.

Many nondestructive evaluation (NDE) methods are already being utilized for structural health monitoring of concrete infrastructures. Indeed, there are already books that discuss the use of NDE methods in evaluating different structures and materials [4,5,6,7]. One common method is the use of NDE based on the elastic wave propagation such as surface wave measurements, ultrasonic pulse velocity (UPV) test, seismic refraction test, acoustic emission test, impact-echo test, etc. Among these methods, the UPV test is prominently used for condition assessment of concrete in the actual structure due to its simplicity in equipment, working principle and data interpretation, and is well-standardized in the test method in many countries in the world [8]. UPV has been widely used in evaluating different concrete conditions. Recently, there have been studies that used UPV to evaluate building materials modified using industrial wastes [9,10]. In the study of Sadowski et al. [9], the results confirmed the potential of using the hybrid ultrasonic-neural method in analyzing the compressive strength of concrete screeds with materials from industrial waste. Another study by Seghir et al. [10] used UPV in evaluating cement-based building materials with marble powder. The results of the research showed that incorporation of marble powder affect the UPV, apparent density, and compressive strength. It was also concluded from the study that UPV depends on both the apparent density and total voids content in cement-based building materials. Other studies have used UPV in evaluating the compressive strength of concrete structures that are in early stages and in very old age [11,12]. The study by Hong et al. [11] correlated the UPV with the compressive strength according to the age. This way, compressive strength of concrete at early stage can be estimated by UPV that may help quality control and quality assurance of concrete during construction. In the study of Saint-Pierre et al. [12], UPV is used to designate concrete quality in old concrete structures. Moreover, it has been demonstrated that UPV is a good indicator of evaluating durability of concrete. Previous researchers established the relationship between UPV and durability indicators of concrete such as permeability, porosity, and water absorption [13,14,15]. The study by Salam et al. [14] concluded that there is a strong correlation between UPV and permeable porosity and water absorption of self-consolidating concretes. It was also shown in the study of Benouis and Grini [13] that there is strong correlation between UPV and porosity for concretes with a water-to-cement ratio greater than or equal to 0.5. The use of the NDT techniques creates one way of reducing the significant budgets, both in cost and in time, incurred when dealing with invasive or destructive techniques in evaluating the mechanical properties of concrete [16].

Previous researchers have established empirical relationships that relate the UPV parameters and static mechanical properties of concrete. Until now, P-wave velocity measurements have been predominantly utilized to evaluate UPV of concrete in the laboratory and the field practice. However, it is, at times, not easy to correctly evaluate mechanical properties from P-wave velocity measurements because of theoretical and practical difficulties [17,18]. For example, it has been shown that the relationship between compressive strength (*f_c_*) and UPV can be affected by various factors such as physical properties of the cement paste, type and size of aggregate, curing conditions, concrete age, and mixture proportions. Therefore, the correlation between *f_c_* and P-wave velocity must be calibrated for each specific concrete mixture proportion. Even if applied to the same concrete mixture, P-wave velocity of concrete could be affected by various parameters such as the confinement conditions, environmental effects and heterogeneity of concrete that are not easily considered in the relationship between UPV and *f_c_*. Moreover, it has been demonstrated by previous researchers that the increasing moisture content (or water saturation level) affects the static properties of concrete and P-wave velocity of concrete in different ways. Some researchers corroborated that the compressive strength decreases as the saturation level increases [1,19,20,21,22,23]. Some researchers [1,20,23,24] observed that the modulus of elasticity increased when the saturation increases while others [25,26] have observed that elastic modulus decreased as the saturation increases. The study of Zhou et al. [25] observed that the elastic modulus increased first then finally decreased as the degree of saturation increased. In contrast, it has been observed that P-wave velocity increases as the saturation level increase in concrete. Cadoni et al. [27] found out that the sensitivity of concrete response is greatly influenced by the amount of free water inside the concrete. Popovics [28] studies the effects of the uneven moisture distribution to the ultrasonic velocities in concrete. In the study, it was observed that P-wave velocities are sensitive to water, thus special care is needed when dealing with P-wave velocities when relating to the compressive strength of concrete.

In contrast, theoretically, shear wave propagation in porous material is not sensitive to water content in pore systems or in confinement conditions of concrete but, until recently, there was a scarcity of available equipment for measurement of shear waves. Regardless of this, there have been experimental results demonstrating that S-wave velocity shows good correlation to mechanical properties of concrete (*f_c_* and *E_c_*) with less sensitivity to other material and environmental effects. Voigt et al. [29] indicated that S-wave reflection is closely related to the level of interparticle bonding irrespective of amount of water in cement paste. An et al. [30] noted that S-wave velocity, regardless of the curing age, curing conditions, and types of aggregate, is strongly associated with compressive strength. Zhu et al. [31] showed experimentally that S-wave velocity in cement paste is not influenced by air content in a range of 0.2%–5.2%, whereas P-wave velocity is too susceptible to air content in a range of 0.2%–2.0%. Ciancio and Helinski [32] exhibited that the S-wave velocity evaluation has been shown to be efficient in assessing the fiber reinforced shotcrete’s compressive strength in field tests due to its reduced sensitivity to the existence pore water. Liu et al. [33] examined the setting and hardening activities of concrete and mortar in the laboratory using ultrasonic S-wave velocity, and confirmed that S-wave velocity was well associated with mortar penetration resistance irrespective of the water-to-cement ratio and test setup. In summary, the S-wave velocity method can evaluate the elastic modulus and concrete compressive strength as an in situ NDE technique. Additionally, recently, commercially available devices for shear wave velocity measurement have been already developed and used to investigate various applications of non-destructive evaluation of concrete in structures [18,34,35,36]. However, it is still difficult to draw a general conclusion due to a lack of experimental studies on evaluating dynamic and static properties of concrete in the different saturation conditions using the S-wave velocity measurements.

Therefore, the general objective of this research is to investigate the effect of water saturation condition to the two different ultrasonic wave velocities (P- and S-waves) and two mechanical properties of concrete (compressive strength and elastic modulus of concrete). Consequently, the study would relate the dynamic and static mechanical properties, particularly to the compressive strength and the static modulus of elasticity of concrete with various saturation levels. Specifically, this research will propose a relationship between S-wave velocity and compressive strength.

## 2. Materials and Method

**Figure 1:**
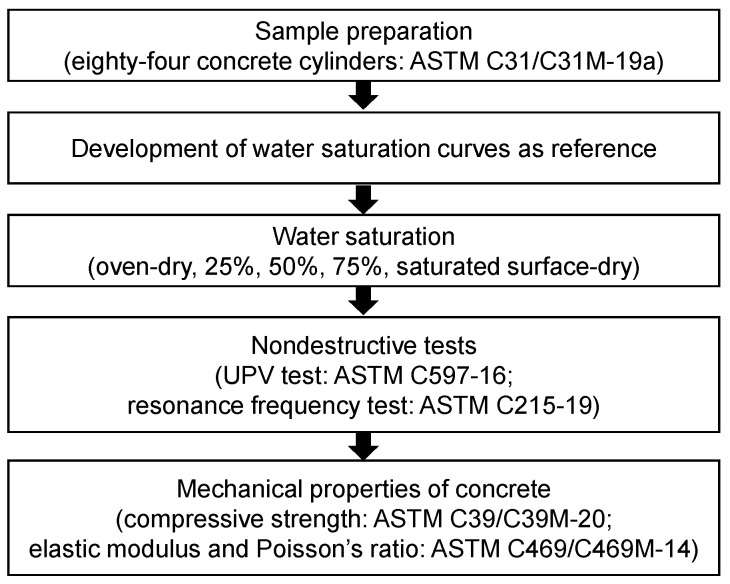
General research flow for this study.

In general, the methodology for this study followed the flowchart shown in Figure 1. Samples were prepared for all the tests that were done for the study. A saturation curve was then developed to use as reference for the tests on saturated concrete cylinders. After which, actual saturation followed, then nondestructive and uniaxial compressive tests were performed. Details of the methodology are explained fully in the subsequent sections.

### 2.1. Sample Preparation

A series of cylindrical specimens, with 200 mm height and 100 mm diameter, was manufactured for the experiment. In this study, the concrete is composed of crushed granite with a maximum size of 25 mm, crushed sand, Type I Portland cement, and additional cementitious materials (slag cement and fly ash). There were different water-to-binder ratios (W/B) of 0.52, 0.35, and 0.26, used in this study, resulting in three different design compressive strengths of 24, 40, and 70 MPa, respectively. The concrete mixtures (referred to as MIX 1, MIX 2, and MIX 3) were manufactured with ready-mixed concrete and the quantities are tabulated in Table 1. Chemical and physical compositions of binders (cement, fly ash, and blast furnace slag cement) are summarized in Table 2. Basic physical properties of chemical admixture (high performance air-entraining agent) used in this study are shown in Table 3. Seventy-five concrete cylinders were prepared in 200 mm (height) by 100 mm (diameter) plastic molds according to ASTM C31/C31M-19a [37]. After being demolded on the next day of casting concrete, the cylinders were water-cured.

### 2.2. Water Saturation

The standard concrete cylinder specimens were subjected to different levels of saturation and moisture content. The saturation curve was initially generated to reach a specific level of saturation. There were five target saturation levels for this study—two standard saturation levels of oven-dry (OD) and saturated-surface dry (SSD), and three other degrees of saturation (25%, 50%, and 75%). The control factors for this procedure were the mix proportion and the immersion time in water. For this method, three specimens from each design mix were used with a total of nine cylindrical specimens. After the specimens were cured under water for at least 150 days, they were put inside the electronic oven for at least 72 h to dry with constant temperature of 105 °C. The mass of the specimens was recorded thirty minutes after they were taken out from the oven. Then, they were placed in small tanks in groups and tap water was added slowly to ensure that the specimens were still submerged in water after the initial absorption of water by the concrete. The mass of the specimens was recorded every 30 min for the first ten hours. The specimens were taken out from the tanks and then excess water was wiped using a wet cloth. After mass was recorded, the cylinders were put back into the water for continuous immersion. After which, mass was recorded every 24 h until the tenth day of immersion time. The total immersion time of the specimens was 240 h. The degree of saturation was calculated using Equation (1).

(1) $$s\;=\;\frac{m_{n}-m_{0}}{m_{w}-m_{0}}\times 100\;\left(\%\right)$$

where s is the water saturation of concrete; $m_{0}$ is the mass of concrete in the OD condition; $m_{w}$ is the mass of concrete in the SSD; and $m_{n}$ is the mass of concrete at the time of measurement for different saturation conditions. When the rate of mass change became smaller than 1 g/h, the concretes were already considered to be in a saturated state [19].

**Figure 2:**
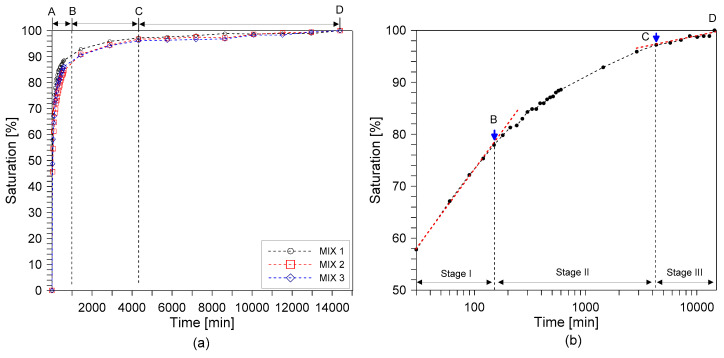
The curve of water saturation with stages in water saturation trend: (**a**) variation of saturation of the three concrete types (MIX 1, 2, and 3) in a linear time scale and (**b**) variation of saturation of concrete MIX 1 in a logarithmic time scale.

Figure 2a shows the saturation curve of the concrete cylinders versus the immersion time in minutes for MIX 1, 2, and 3. Figure 2b shows the variation of saturation with time in a logarithmic scale. There are three defined stages in the water saturation trend—the log linear stage (AB), the non-linear stage (BC), and the approximate saturation stage (CD) with log linear relation. In the first stage (for the first 600 min), all concrete cylinders show a fast absorption rate, with MIX 1 having the fastest rate followed by MIX 3 and MIX 2. In this stage, water saturation in concrete is controlled by diffusion of surface water towards the inside of concrete in which water saturation increased log-linearly with increasing time (see Figure 2b). After then, the saturation rate of MIX 1, 2, and 3 gradually decreased, which was defined as stage 2 in this study. In the third stage (after 10,080 min), the degrees of saturation of Mixes 1, 2, and 3 reached 98.7%, 98.6%, and 98.3%, respectively. At this time, the rate of absorption had already slowed with a mass change of less than 1g/h.

The saturation curve initially developed from this procedure, shown in Figure 1, was then used for achieving the saturation degree of the specimens tested for this study. Some variations were observed in the measured saturation levels of concrete cylinders (see Table 4), mainly due to heterogeneous features of concrete. The actual saturation during testing was the approximation of the target saturation from the reference curve. However, the actual values were very close to the target saturation levels in this study, which demonstrate the effectiveness of the procedure in this study.

### 2.3. Stress Wave Velocity Measurement

In this section, the variables for the experiment were the degree of saturation and the design mix proportions. The materials and experimental groups were similar with those used in the development of water saturation curve explained in the previous section. There were a total of fifteen experimental groups set up for this measurement. Five specimens from each design mix, for each saturation level, were used for testing giving a total of 75 specimens. Moreover, the frequencies of the transducers used for this study were 40 kHz and 50 kHz, which gave a wavelength less than the length of the cylinders used for the experiment.

#### 2.3.1. Ultrasonic Pulse Velocity

**Figure 3:**
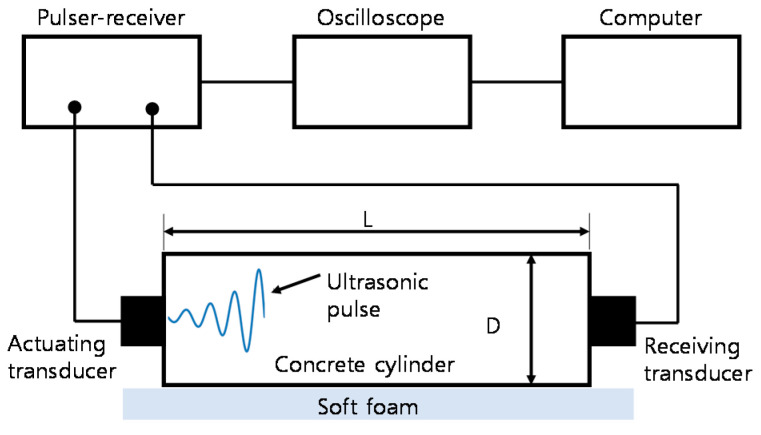
Test setup for ultrasonic pulse velocity measurements.

The P-wave velocity (constrained compressive velocity) of concrete cylinders with five different saturation levels was evaluated using the standard test method following ASTM C 597-16 [38]. The study used a pair of transducers, which can transmit and receive ultrasonic pulses of about 50 kHz (see Figure 3). Using a pulse-receiver (Panametrics 5077 PR), the source transducer was driven by a 200 V square pulse having a duration of 10 µs. The transient stress waves produced by the source sensor, propagated in the concrete, and were gauged by the receiving sensor. The received signal was digitized, with a total signal length of 0.001s and at a sampling rate of 10 MHz, by a high-speed digital oscilloscope (NI-PXI 5101, Austin, TX, USA). The digitized data were moved to a computer for postprocessing and data storage.

**Figure 4:**
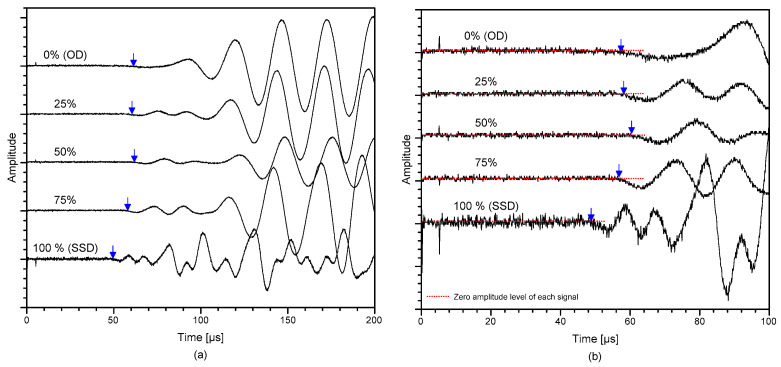
Typical time signals of ultrasonic pulse waves (P-wave) propagating through saturated concrete cylinders: (**a**) ultrasonic waves at different saturation levels and (**b**) enlarged ultrasonic signals around the first arrival of the wave (P-waves) shown in Figure 4a.

Figure 4a shows the typical time signals measured from concrete cylinders from MIX 1. Ultrasonic pulse signals measured from concrete cylinders under five saturation conditions are presented in the figures. Furthermore, Figure 4b shows the enlarged ultrasonic signals around the first arrival of the P-waves shown in Figure 4a. Note that DC component of ultrasonic signals was removed in the frequency domain to adjust the zero-signal level to zero at the y-axis. The P-wave velocity was estimated by dividing the travel distance, which is the length of the specimen being tested, by the travel time of the wave as shown in the equation below,

(2) $$V_{P}=\;\frac{d}{\left(t_{a}-t_{d}\right)}$$

where *V_p_* is the velocity of wave propagation, *d* is the distance between transducers, *t_a_* is the time of first wave arrival, and *t_d_* is the delay time, calculated during calibration of the probes. Delay time was determined when time for the first arrival wave was registered when the two transducers were positioned against each other. From the measured ultrasonic signals, the arrival of transient stress waves through cylinders was calculated using the modified threshold process [39]. In this approach, an estimated arrival time was first acquired using the typical threshold method in the previous studies [40]. Then, a precise time of arrival was determined by fitting a line to the signal data. The P-wave travel time was then defined by the intersection of the fitting line and the measured zero-signal stage.

**Figure 5:**
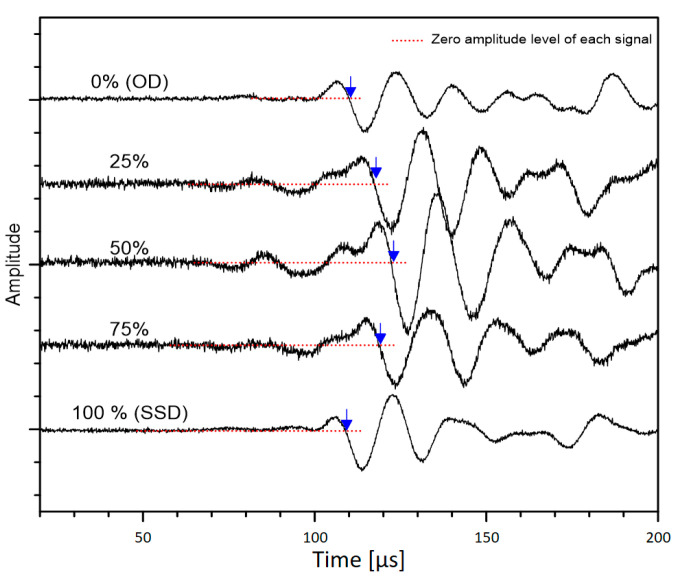
Typical time signals of ultrasonic pulse waves (shear wave propagating through concrete cylinders at different saturation levels, measured by a pair of the dry contact S wave transducers.

The S-wave velocity of concrete, V_S,UPV_, was measured using the P-wave velocity method depicted in the previous section but using a pair of S-wave transducers (40 kHz) (40 kHz dry-point shear wave transducer produced by Proceq, Schwerzenbach, Switzerland). The S-wave transducer had a diameter of 84 mm and a length of 114 mm and a weight of 340 g, which is portable and can be operated by a single person. It has an eight dry point shear wave sensor array, which does not require extra coupling agent (such as a viscous and sticky coupling gel), which minimizes the effect of coupling conditions between concrete surface and the transducer. The dry contact feature of shear wave sensors significantly ensured reliable and consistent data acquisition and significantly improved the test speed. Typical impulse signals produced by a source transducer and an ultrasonic wave measured by a receiving transducer for the S-wave velocity measurement is shown in Figure 5. Similar to the P-wave velocity measurement method, the first arrival time of the S-wave was also determined by the modified threshold method. However, unlike P-waves shown in Figure 4, due to the interference between direct P- and S-waves, precise detection of the first arrival time of the S-waves is often difficult. Even using S-wave transducers, low amplitude P-wave components still appear in the time domain along with the S-wave components. In this study, the first arrival of the S-waves was defined as the intersection of the fitting line to the first negative component of the S-wave and the calculated zero signal level, shown as red dashed line in Figure 5. To be clear, the first signal with low amplitude was assumed to be from P-waves.

#### 2.3.2. Resonance Frequency

**Figure 6:**
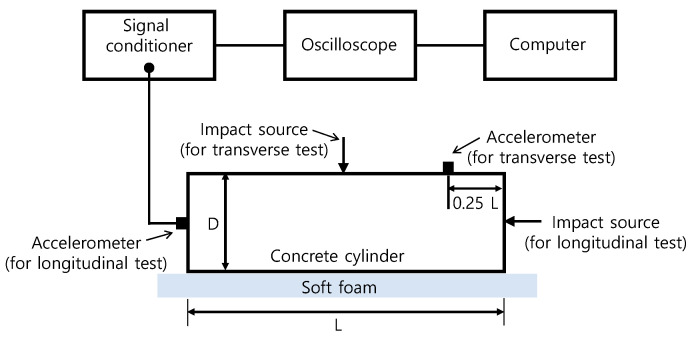
Test setup for resonance frequency tests.

A free–free resonant (FFR) frequency test was used in this study to measure the wave velocities through a concrete cylinder specimen. The FFR test set up for this study following ASTM C215-19 [41] is shown in Figure 6. To replicate fully free boundary conditions, concrete cylinder samples were laid flat above a soft polyurethane foam. For generation of the incident stress wave in concrete cylinders, a steel ball with a diameter of 12 mm was used. The steel ball was efficient in producing broadband frequency signals from very low to 20 kHz, which covers the resonance tests’ frequency range in this research. The dynamic response of the cylindrical specimens was evaluated by an accelerometer (PCB352C33, PCB Piezotronics, Depew, NY, USA), attached to the concrete specimen in accordance with the ASTM C215-19 [41], with a resonance frequency of around 50 kHz and 5% frequency range (from 0.5 Hz to 10 kHz) [42]. The obtained signals using the accelerometer adjusted using an NI-USB 6366 oscilloscope at a sampling frequency of 1 MHz and were sustained using a signal conditioner (PCB 482C16, PCB Piezotronics, Depew, NY, USA). The acquired time signals were transformed, using the FFT (fast Fourier transform) algorithm, to the frequency domain. The resonance frequencies of the samples were shown as predominant peaks in the amplitude spectrum. The highest frequency was considered the fundamental resonance frequency for transverse *f_Tr_* and longitudinal *f_L_* modes [43].

**Figure 7:**
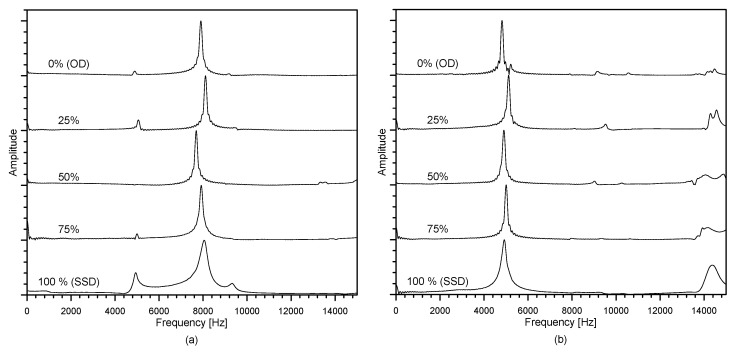
The variations of spectral amplitudes of dynamic response of MIX 1 concrete cylinders in: (**a**) the fundamental longitudinal mode and (**b**) the fundamental transverse mode.

Figure 7 shows the typical spectral amplitudes of the dynamic response of concrete cylinders measured from the transverse and longitudinal resonant tests. For this study, the wave velocities were computed using Equations (3) and (4), respectively.

(3) $$V_{S,RF}=\;2L\frac{f_{Tr}}{K_{G}}$$

(4) $$V_{C}=\;2L\frac{f_{L}}{K_{E}}$$

where *f_Tr_* and *f_L_* are the required resonance frequencies for transverse and longitudinal modes, respectively, determined by the resonance test; *L* is the length of the cylindrical specimen (see Figure 6) and *K_G_* is a correction factor adapting the transverse resonance frequency to the torsional resonance frequency for a 100 mm × 200 mm concrete specimen (D/L = 0.5), as follows [44]:

(5) $$K_{G}=\;0.337\nu_{d}+0.8833$$

*K_E_* is a correction factor to address the scattering effect of stress waves in a 200 mm × 100 mm concrete specimen (D/L = 0.5, where D is the diameter of a concrete cylinder) as follows [44]:

(6) $$K_{E}=\;-0.095\nu_{d}+1.0125$$

where $\nu_{d}$ is the dynamic Poisson’s ratio of concrete. It should be noted that the dynamic Poisson’s ratio used in Equations (5) and (6) were calculated from the wave velocities measured using the UPV test. This was done to account for the change in the density of the material as the mass of the specimens increased because of the absorption of water.

#### 2.3.3. Measurement of Mechanical Properties

**Figure 8:**
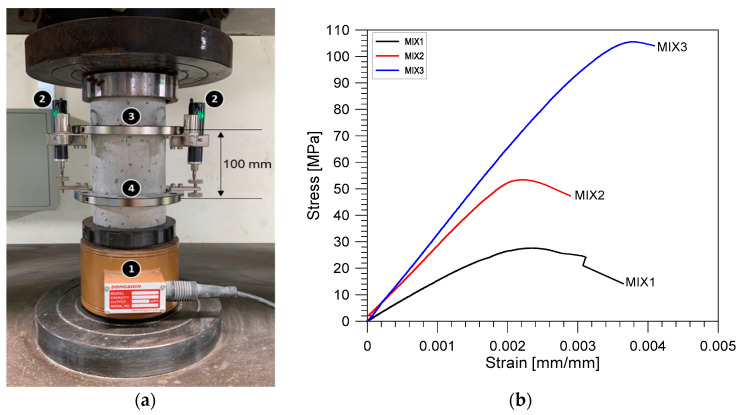
(**a**) Test setup for the uniaxial compressive test for measurements of compressive strength and static modulus of elasticity of concrete cylinders and (**b**) typical stress and strain relations calculated from the concrete cylinders with MIX 1, 2, and 3 in the fully dry conditions (oven-dry (OD)).

The compressive strength and elastic modulus of the cylindrical specimens were determined using a universal testing machine (UTM, KST, Busan, Korea), with a capacity of 2000 kN, after the measurement of stress wave velocities. The specimens were at the age of around 226–263 days at the time of testing. The compressive strengths and elastic moduli were measured under displacement control in accordance with ASTM C39/C39M-20 [45] and ASTM C469/C469M-14 [46], respectively. Uniaxial compressive tests were done with an axial movement rate of 2 mm/min. It was confirmed from preliminary tests that the test speed in this study was equivalent to 0.28 MPa/s in the elastic regime of concrete. Compressive loads employed to the surface of concrete cylinders were measured by a load cell with a capacity of 2000 kN, ① in Figure 8a. Deformations were evaluated by employing two sets of extensometers fastened to two fixed frames, ② in Figure 8a. It has two aluminum rings with screws for fastening the specimen, ③ and ④ in Figure 8a. The top and bottom aluminum rings has a spacing between them equal to 100 mm, which serves as the gauge length (*L_0_*) to determine axial strain from the evaluated deformations. The load and deformation data measured by the load cell and the extensometers were digitized by a data acquisition equipment (DEWE43A, DEWESoft, Trbovlje, Slovenia) with a sampling frequency of 100 Hz. Figure 8b shows typical stress and strain curves measured from the MIX 1, 2, and 3 at the fully dry conditions (OD). Instantaneous axial stress ($\sigma_{i}$) and strain ($\varepsilon_{i})$ were calculated from the instantaneous load ($P_{i}$) and axial deformation data ($L_{i}$) using Equations (7) and (8), respectively.

(7) $$\sigma_{i}=\frac{P_{i}}{A}$$

(8) $$\varepsilon_{i}=\frac{L_{i}}{L_{0}}$$

The concrete modulus of elasticity is characterized as the chord modulus from the stress–strain curve and is determined using Equation (9), which is called the static modulus of elasticity of concrete in this study.

(9) $$E_{s}=\frac{0.4{F\prime }_{c}-\sigma \left(\varepsilon_{1}\right)}{\varepsilon_{2}-\varepsilon_{1}}$$

where $\varepsilon_{1}$ is the longitudinal strain of 0.00005 and $\varepsilon_{2}$ is the longitudinal strain corresponding to the 40% of maximum stress (${F^{\prime }}_{c}$). In this study, the first and second points were established by a linear regression of the local data in the calculated stress–strain curves.

## 3. Results and Discussion

### 3.1. Variation of Stress Wave Velocities with Water Saturation Level

**Figure 9:**
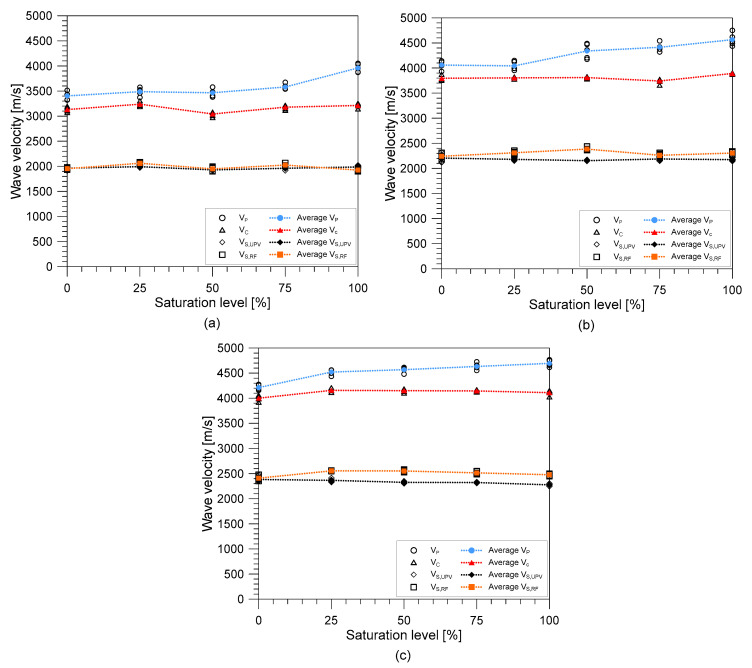
Variation of the three wave velocities (constrained compressive wave velocity, V_P_, unconstrained wave velocity V_C_, and shear wave velocity, V_S,UPV_ and V_S,RF_) of submerged concrete as the water saturation increases measured from concrete with: (**a**) MIX 1, (**b**) MIX 2, and (**c**) MIX 3.

Figure 9 illustrates the variation of the stress wave velocities (unconstrained wave velocity (*V_C_*), two shear wave velocities from the UPV test (*V_S,UPV_*) and resonance frequency test (*V_S,RF_*), and constrained compressive wave velocity (*V_P_*)) from the submerged concrete as the water saturation increases from 0% (OD condition) to 100% (SSD condition) with intervals of 25%. Equations (2)–(4) were used to calculate *V_P_*, *V_C_*, and *V_S,RF_*, respectively, while *V_S,UPV_* was measured using the same concept of Equation (2) using the time domain signal of shear wave measurement from the UPV test. Overall, the unconstrained wave velocity (*V_C_*) and the constrained compressive wave velocity (*V_P_*) increase as the water saturation of concrete increased. All mixes of concrete cylinders exhibited a gradual increase of *V_P_* as the water saturation level increased from 25% to 75% with MIX 1 increasing by about 5.2%, MIX 2 by 7.7% and MIX 3 by around 10%. Then an abrupt increase in wave velocity was observed from a saturation level of 75% until approximate full saturation by about 16.3% and 12.5% for MIX 1 and MIX 2, respectively. MIX 3 continued to exhibit a gradual increase of about 11.5% at approximate fully saturated state. For *V_C_*, all mixes generally exhibited a gradual increase in velocity as the level of saturation became higher with an average increase of about 3–5.7% of the velocity when fully dried. In contrast, the values of the shear wave velocities from both the UPV and resonance tests were almost constant as the concrete cylinders become more saturated. MIX 2 and MIX 3 showed a decrease (1.5% and 4.5%, respectively) in shear wave velocity (*V_S,UPV_*) as the cylinders became fully saturated while MIX 1 demonstrated a very slight increase of about 1.3%. In the case of V_S_, MIX 2 and 3 showed an increase in the values by around 1.8% and 2.8%, respectively, while MIX 1 showed a decrease in value by about 1.5%.

**Figure 10:**
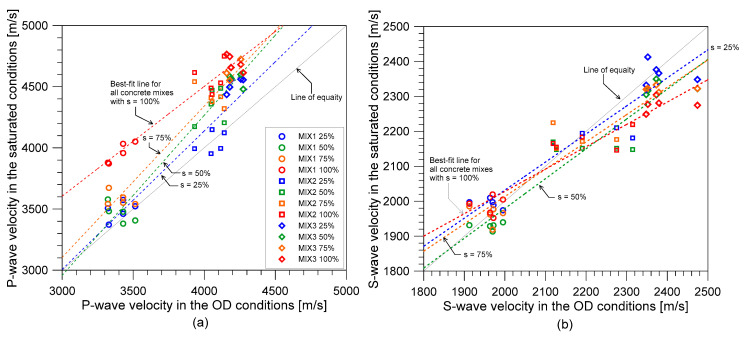
Relationship of stress wave velocities of the fully dry concrete and the saturated concrete for (**a**) P-wave velocities and (**b**) S-wave velocities.

Figure 10a,b illustrates the relationship of P- and S-wave velocities, respectively, of the fully dry concrete (OD conditions) and the saturated concrete with saturation levels of 25%, 50%, 75%, and 100%. Data set from the MIX 1, 2, and 3 were represented by an open circle, square, and diamond symbols, and different saturation levels of 25%, 50%, 75%, and 100% were represented as blue, green, orange, and red, respectively. For comparison, approximate equations relating the wave velocities of the saturated concrete and the fully dry concrete were established by linear regression analysis. The best-fit lines for MIX 1, 2, and 3 with the saturation levels of 25%, 50%, 75%, and 100% are presented in the figure as dash lines with blue, green, orange, and red, respectively. Figure 10a shows that P-wave velocities are clearly affected by the level of water saturation in concrete cylinders as the data points become further away from the line of equality. In contrast, shear wave velocities appear to be less sensitive to the level of water saturation compared to the P-wave velocities.

The effect of saturated water in concrete can be explained by Biot’s theory [47]. The properties of each constituent material could be determined from the solid constituent, the elastic properties (density and moduli) and the porosity of the fluid, and framework of the material. In the case of shear wave velocity in saturated concrete, the shear modulus did not change. However, material density increased because of the addition of water, giving a slight decrease in shear wave velocity as the concrete becomes more saturated. However, in the case of compressive waves, it is also dependent on the bulk modulus of the material. As the material becomes saturated, bulk modulus tended to increase, thereby, increasing the compressive wave velocities (*V_P_* and *V_C_*) as well. 

### 3.2. Poisson’s Ratio of Concrete at Different Saturation Levels

**Figure 11:**
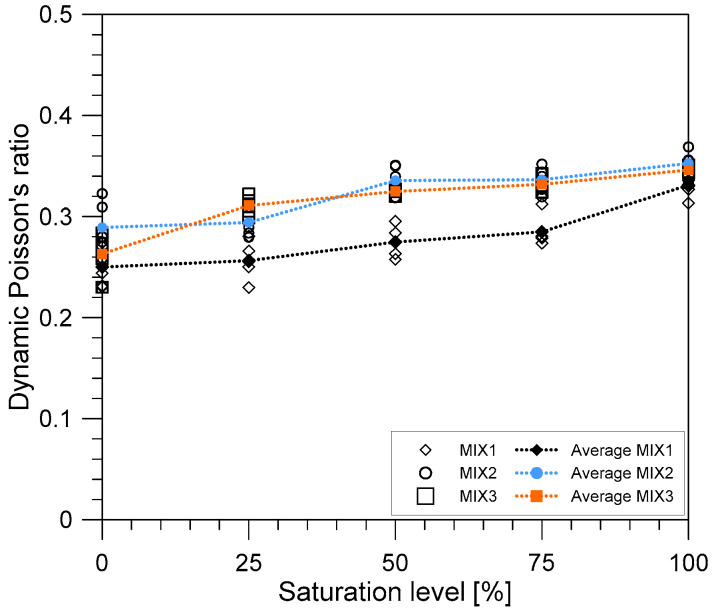
Variation of dynamic Poisson’s ratios with the water saturation level of concrete.

The measured wave velocities of concrete give estimated values of the dynamic Poisson’s ratios. The variation of the dynamic Poisson’s ratios of concrete with respect to the saturation condition of the concrete is illustrated in Figure 11. In this research, the dynamic Poisson’s ratio was calculated using the following equation,

(10) $$\upsilon_{d}=\frac{\gamma^{2}-2}{2\gamma^{2}-2}$$

where $\upsilon_{d}$ is the dynamic Poisson’s ratios measured from the P- and S-wave velocities from the UPV test and *γ* is the ratio of the P-wave and S-wave velocities from the UPV test. When the concrete cylinders are completely dried, the dynamic Poisson’s ratios are in the range of 0.25–0.35 across all mixes. These values are higher than the typical reported values of Poisson’s ratio. One of the possible reasons is the difficulty in picking up the accurate time for the arrival of the first wave, especially for shear wave velocities. Higher values of dynamic Poisson’s ratio in this study may be attributed to the lower values of measured shear wave velocity according to the criteria of determining the first arrival of S-wave velocity in this study. When the saturation level increased from the oven-dry conditions (0% saturation) to the SSD conditions (100% saturation), the dynamic Poisson’s ratio increased by 32.36%, 22.02%, and 31.56% for the MIX 1, 2, and 3, respectively. The values of the dynamic Poisson’s ratio were used to evaluate the dynamic elastic modulus of concrete in the next section. 

### 3.3. Static and Dynamic Elastic Modulus of Concrete at Different Saturation Levels

**Figure 12:**
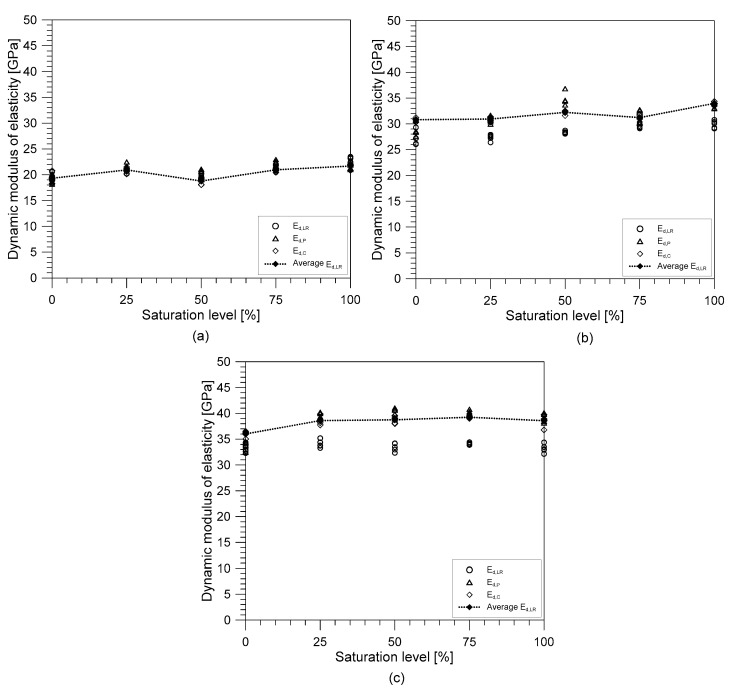
Variation of dynamic elastic modulus of concrete with increasing saturation level for: (**a**) MIX 1, (**b**) MIX 2, and (**c**) MIX 3. Note that the dynamic elastic moduli, *E_d,P_* and *E_d,S_*, were calculated with a Poisson’s ratio from Section 3.2.

The variation of dynamic elastic modulus of concrete with an increasing degree of saturation is illustrated in Figure 12. In this research, the dynamic modulus of elasticity was determined using the equation specified in ASTM C215-19 [41] by measuring the fundamental longitudinal resonance frequency (*f_L_*) as follows,

(11) $$E_{d,LR}=DMf_{L}^{2}$$

where *D* is a constant reliant on the specimen’s dimensions and Poisson’s ratio of concrete specimens (equal to $5.093\frac{L}{d^{2}})$ for cylindrical specimens in N·s^2^ (kg·m^2^); *M* is the mass of the specimen in kg; L is the length of the specimen in m; and d is the diameter of the specimen in m. In this study, $E_{d,LR}$ is called the resonance modulus. In Figure 12, each *E_d,LR_* value from a concrete cylinder is presented as an open circle and the average of *E_d,LR_* values obtained from concrete cylinders for the same concrete mixture proportion and saturation condition are presented as dash lines. The resonance modulus slightly increased as the saturation level also increased. At the full saturation level (100%), the increase in the dynamic elastic modulus was about 7.1%–12.3%, with the highest increase in MIX 1, followed by MIX 2 then MIX 3. From 0 to 25%, the increase was very minimal for MIX 2 with the rate of 0.4% while for MIX 1 and 3, the rates were 8.3% and 7.2%, respectively. The values of the dynamic elastic modulus from 25% to 75% were fluctuating although their coefficients of variation were within the reasonable values ranging from 1.6% to 3.5% for Mix 1, 1.1% to 3.0% for MIX 2, and 0.5% to 2.1% for Mix 3. Note that another resonance modulus based on the measured transverse resonance frequency (*f_TR_*) ($E_{d,TR}$) described in ASTM C215-19 [41] showed very good agreement with $E_{d,LR}$, with a range of error within 1–2%, but it was not shown in the Figure 12.

For comparison, two additional dynamic elastic moduli were calculated using Equations (12) and (13) using the values of P- and S-wave velocities measured by ultrasonic pulse velocity measurements, which are called the velocity moduli.

(12) $$E_{d,P}=\frac{\rho \left(1+\upsilon_{d}\right)\left(1-2\upsilon_{d}\right)}{\left(1-\upsilon_{d}\right)}V_{P}^{2}$$

(13) $$E_{d,S}=2\rho \left(1+\upsilon_{d}\right)V_{S}^{2}$$

where *E_d,S_* and *E_d,P_* are the dynamic moduli of elasticity of concrete based on *V_S,UPV_* and *V_P_*, respectively; and $\upsilon_{d}$ is the concrete’s dynamic Poisson’s ratio. In Figure 12, the two velocity moduli are shown for each mix design. The value of Poisson’s ratio used for the calculation of the dynamic modulus of elasticity was equal to the Poisson’s ratio evaluated at Section 3.2. Of the three dynamic elastic moduli, only the two velocity moduli (*E_d,P_* and *E_d,S_*) were dependent on the value of the Poisson’s ratio. As discussed in Section 3.2, the level of saturation affects the Poisson’s ratio: as the level of saturation increases, the value of Poisson’s ratio also increases. It might be expected that this would also greatly increase the dynamic modulus, *E_d,P_*. However, as can be observed from the graph, there is only a minimal effect of the Poisson’s ratio to the said parameter. Meanwhile, dynamic modulus *E_d,LR_* also showed the same behavior when the saturation level increased. From this assessment, it can be inferred that Poisson’s ratio does not greatly affect the values of the dynamic elastic moduli. Therefore, it can be deduced from this observation that the saturation condition greatly affects the values of the dynamic Poisson’s ratio and consequently, the dynamic elastic modulus but not as much as with the Poisson’s ratio.

**Figure 13:**
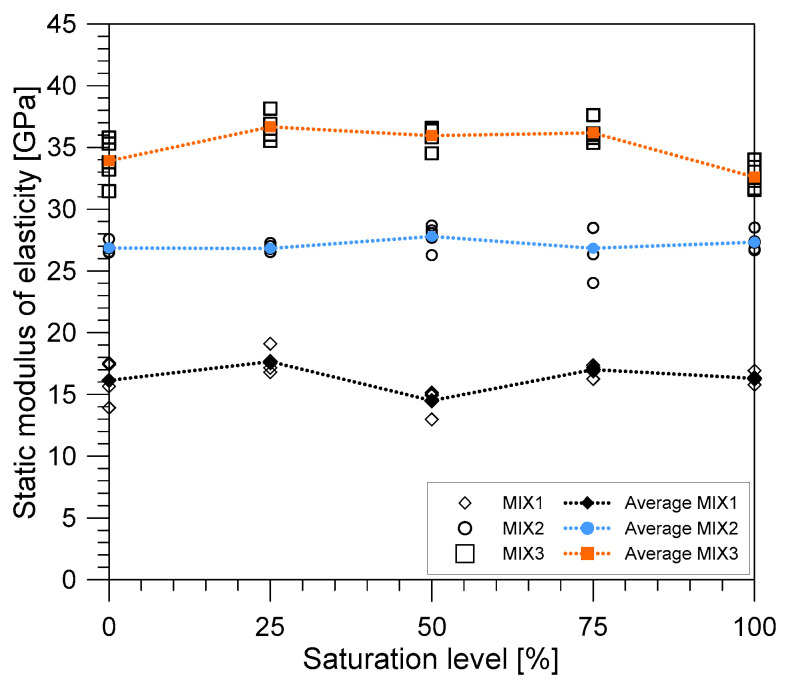
The variation of static modulus of elasticity of concrete with saturation condition.

Figure 13 shows the variation of the static elastic modulus of the concrete with the degree of saturation. The values of the static elastic modulus for MIX 1 and 2 increased slightly by about 1.1% and 1.7%, respectively, as the degree of saturation increased. On the other hand, the static modulus of elasticity for MIX 3 decreased by around 3.9% as the saturation level increased. The differences in the static elastic moduli of the different concrete mixture proportions may be attributed to the inherent property of concrete as an inhomogeneous multiphase material. The elastic behavior of the concrete is determined by properties of various concrete constituents, which include the materials’ densities, transition region properties, total volume percentage [26], pore geometry, contained water, and matrix elasticity [48], among others. When concrete is in the process of drying, the moisture in the initial microcracks of the transition zone and in the pores evaporated, which would eventually affect the volume of the material, which could then generate deformations. On the other hand, saturated concretes or wet concretes exhibited slight variation between bulk modulus of the free water in the concrete matrix in the pores. Moreover, the presence of the C-S-H gels creates an increase in the elasticity modulus. The decrease in the static modulus of elasticity of MIX 3 might be explained by the phenomena during drying when microcracks developed in the transition region of the concrete [26]. 

### 3.4. Relationship between Dynamic and Static Elastic Moduli

**Figure 14:**
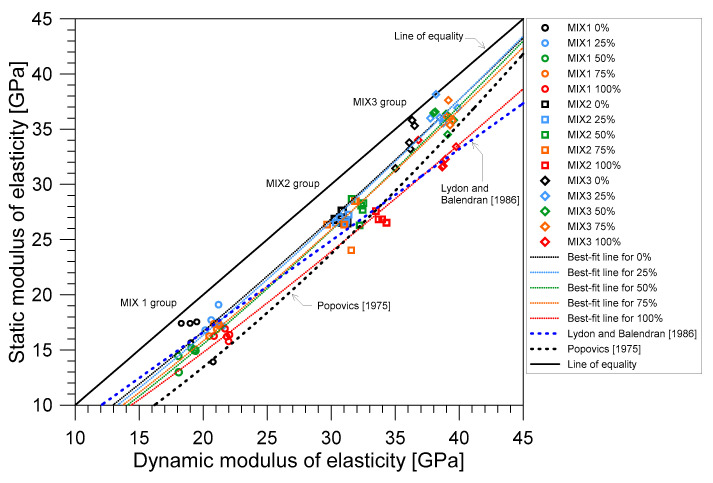
The relationship between static and dynamic elastic moduli of water-saturated concrete.

Figure 14 shows the relationship between the static and dynamic elastic moduli of concrete saturated with water. The dynamic modulus of elasticity was computed using Equation (11), in accordance with ASTM C215-19 [41]. Overall, the static modulus of elasticity is lower than that of the dynamic elastic modulus in the whole range of saturation level, from completely dry (0% saturation) to approximate fully saturated conditions (100% saturation).

It was observed that the relationship between dynamic and static elastic moduli of concrete was affected by water saturation levels in concrete. For example, at the completely dry state, the static modulus of concrete was about 83% of the dynamic modulus for MIX 1, 87% for MIX 2, and 94% for MIX 3. At the fully saturated condition, the difference between the static and dynamic elastic moduli increased. At 100% saturation, static elastic modulus was about 75% of the dynamic elastic modulus for MIX 1, 80% for MIX 2, and 84% for MIX 3. As determined by the experimental aspects of this study, the equation relating the static and dynamic elastic moduli of concrete saturated with water was attained by non-linear regression using the power function relationship:

(14) $$E_{d}=aE_{c}^{b}$$

where $E_{d}$ and $E_{s}$ are the dynamic and static elastic moduli of concrete saturated with water in GPa and *a* and *b* are the best-fit constants according to regression analysis performed in the curve fitting toolbox in MATLAB using the nonlinear least squares method. The curve fitting analysis was done separately for each saturation condition across all mixes. The parameters of the best-fit curves for the experimental data of this study are summarized in Table 5. As can be seen from Figure 14, the best-fit curves almost coincided with each other except for the curve representing the SSD condition (100% saturation). However, the difference was only little between the curve for the SSD condition and those for the partially saturated conditions. Therefore, it can be said that the saturation condition did not greatly affect the relationship between static and dynamic elastic moduli.

Figure 14 also compares the experimental data with the empirical models developed by other researchers [49,50] at room temperature. Lydon and Balendran [49] recommended a linear relationship between the static and the dynamic elastic modulus with an equation described by

(15) $$E_{s}=0.83E_{d}$$

while Popovics [50] recommended a more general relationship for normal and lightweight density concrete:

(16) $$E_{s}=446.09\frac{E_{d}^{1.4}}{\rho }$$

where ρ is the density of the hardened concrete in kg/m^3^. The equation proposed by Popovics [50] takes into account the effect of the concrete density. Note that the relation based on the Popovics equation (Equation (16)) in Figure 14 was calculated by using the concrete density of 2200 kg/m^3^. In general, comparing the two previous equations, the equation proposed by Lydon and Balendran [49] agrees better with the MIX 1 and MIX 2 data in the oven-dry and partially saturation concrete than the Popovics equation. In contrast, the Popovics equation was more consistent to the data from concrete cylinders in the surface-saturated dry conditions. 

### 3.5. Compressive Strength

**Figure 15:**
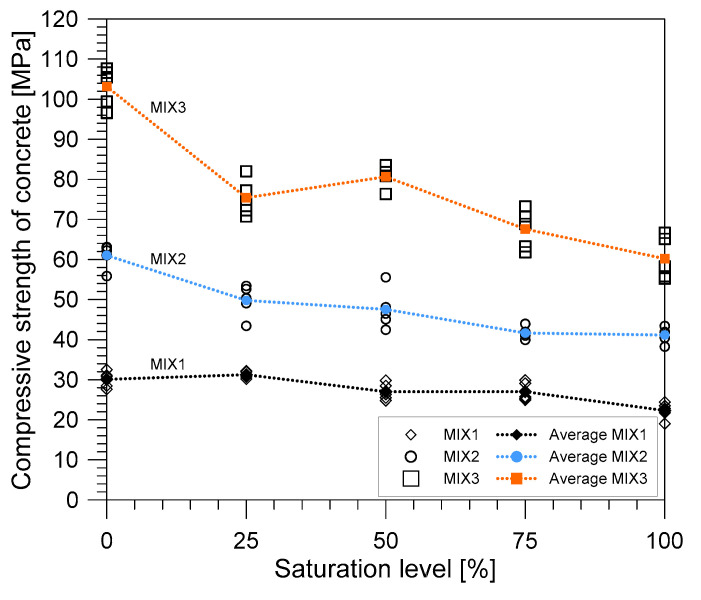
The variation of compressive strength of the different mix proportion with the degree of saturation.

Figure 15 shows the variation of the compressive strength with the degree of saturation of the three different mixture proportions. For the three mixture proportions, compressive strength of concrete decreased with increasing the saturation level. At the fully saturated state, MIX 1 registered a reduction of about 25.9% of the compressive strength from the completely dry state; MIX 2 registered a 32.5% reduction; and, finally, MIX 3 recorded a 41.6% reduction. From the results of this study, it can be inferred that concretes with a lower water-to-binder ratio (MIX 2 and MIX 3) is more sensitive to the effects of water saturation as they registered higher reduction magnitude than that of concrete with higher water-to-binder ratio (MIX 1). This observation is in contrast to the study conducted by Zhang et al. [19], which suggested that concrete mixes with a high water-to-cement ratio might have higher sensitivity to water saturation than those of concrete with a lower water-to-cement ratio. However, in the case of this study, MIX 3 has the lowest water-to-cement ratio among the three concrete mix proportions but it recorded the highest reduction in strength. This observation might suggest that the water-to-cement ratio is insufficient to completely understand the effect of water saturation on the properties of concrete. The decrease in strength can be explained by several factors as explained by previous studies [2,19,21,26,43,51,52,53]. One interpretation is from the standpoint of energy [19,21,23,51,53]. When water enters concrete, it causes the gel elements to part that eventually lessen the van der Waals forces. These forces affect the specific surface energy, which is reduced by the presence of the free water, which acts as a wedged affected by volume deformation of the concrete. The presence of the free water also depends on the porosity of material, which then depends on the materials that constitute the concrete. It is suggested to further investigate the effect of water to the porosity of concrete to further understand this phenomenon.

**Figure 16:**
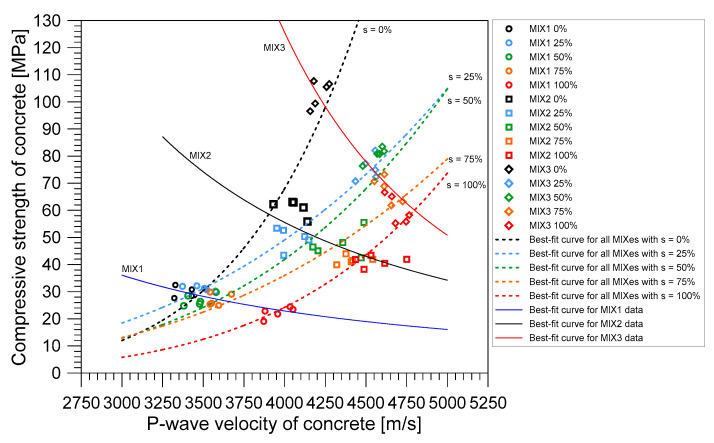
Relationship between P-wave velocity and the compressive strength of saturated concrete.

Figure 16 shows the relationship between compressive strength (*f_c_*) and P-wave velocity (*V_P_*) of concrete cylinders made with the three different mixture proportions (MIX 1, 2, and 3) at the five different water saturation conditions (s = 0, 25, 50, 75, and 100%). Consistent with the observation from Popovics [28], the relationship between *f_c_* and *V_P_* was highly sensitive to the saturation condition of concrete. Consequently, there are many relationships that can be formed between *f_c_* and *V_P_*. For the fixed saturation level of concrete, the concrete made with lower water-to-binder (W/B) resulted in higher values in both *f_c_* and *V_p_*. For comparison, the best-fit curves relating the *f_c_* and *V_p_* of concrete at the saturation levels of 0, 25, 50, 75, and 100% are shown as dashed lines with black, blue, green, orange, and red dashed lines, respectively. In contrast, for the fixed concrete mixture, the water saturation level of concrete affected *V_p_* and *f_c_* in different ways: as increasing water saturation level, *V_P_* increased while *f_c_* decreased. The best-fit curves relating the *f_c_* and *V_p_* of concrete with MIX 1, 2, and 3 with various saturation levels from 0% to 100% in intervals of 25% are shown as solid lines with blue, black, and red lines, respectively, in Figure 16. Therefore, special cares are needed to evaluate compressive strength of concrete with various water content (or saturation conditions) using P-wave velocity measurements in the laboratory and the field practice. 

**Figure 17:**
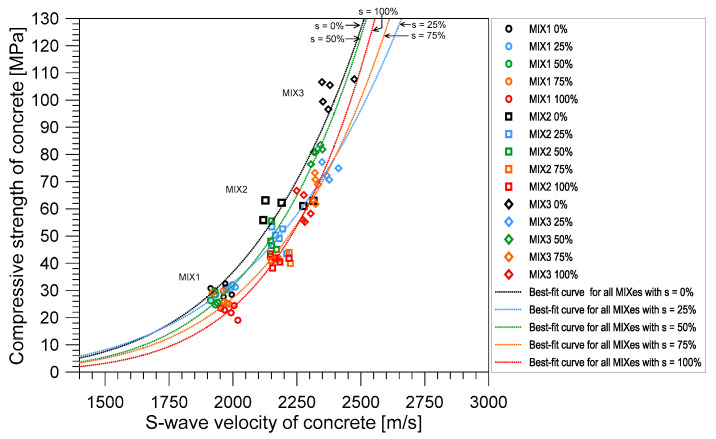
Relationship between shear wave velocity and the compressive strength of saturated concrete.

In contrast, Figure 17 shows the relationship between the shear velocity (*V_S,UPV_*) measured from the UPV test and the compressive strength (*f_c_*) of water saturated concrete. Overall, *V_S,UPV_* increased as *f_c_* increases from MIX 1 to MIX 3. For comparison, the best-fit curves relating the *f_c_* and *V_S,UPV_* of concrete at the saturation levels of 0, 25, 50, 75, and 100% are shown as dashed lines with black, blue, green, orange, and red dashed lines, respectively, in Figure 17. It was noticed that the relationship between *V_S,UPV_* and *f_c_* had a minimal change as the concrete becomes saturated and this observation is true for all mixes. As stated in the study of Lee et al. [17], in theory, shear waves travel perpendicular to the direction of propagation; therefore, they can move through solid material with enough shear rigidity. As a result, movement of shear waves is not affected by fluids in porous materials. However, as shear wave is affected by the density of the material, addition of water can minimally affect its values. In this study, a general equation was derived to relate *f_c_* and *V_S,UPV_* was determined by the nonlinear exponential function as follows,

(17) $$f_{c}^{\prime }=ae^{bV_{S,UPV}}$$

The regression analysis was performed in the curve fitting toolbox of MATLAB using the nonlinear least squares method. The best-fit curve was attained at *a* = 0.1445, *b* = 0.0027 with the least R-square equal to 0.867. The RMSE between the predicted compressive strength based on the recommended equation and the measured compressive strength from the experiment was 9.3 MPa. The value of RMSE was about 40%, 25%, and 15% of the design compressive strength of concrete MIX 1, 2, and 3, respectively. The considerable level of RMSE in this study could be attributed to several experimental uncertainties associated with compressive strength of concrete (see Figure 15), ultrasonic shear wave measurements described in Section 2.3.1 and heterogeneity of concrete. In addition, the effect of water saturation was still influential to evaluate compressive strength of concrete using shear wave velocity measurements. Finally, it is recommended that more study should be done, with more data, to increase the reliability of the equation relating the shear wave velocity and the compressive strength of concrete. 

## 4. Conclusions

This experiment was conducted to investigate the effect of the water saturation to the dynamic and static mechanical properties of concrete. Three different mix proportions of concrete were investigated with five different degrees of saturation from completely dry to approximate fully saturated conditions. The nondestructive test methods used in the investigation of the dynamic properties of concrete were the ultrasonic pulse velocity test and the resonance test. Detailed below are the conclusions attained in this study:

1. The effect of the saturation condition was more evident on the P-wave velocity than in S-wave velocity. The contained compressive wave velocities, from the UPV test, and the unconstrained wave velocity, from the resonance test, increased as the saturation level also increased. S-wave velocities measured from both the UPV and resonance methods exhibited slight changes as the saturation condition increased. This shows that S-wave velocities were less sensitive to the effect of water saturation than the P-wave velocities. This observation could confirm that the use of S-wave velocity to estimate some properties of in-situ concrete was more reliable than the use of P-wave velocity because P-wave velocity was affected by many environmental factors.
2. It is expected that the shear wave velocities will slightly decrease because of the effect of water saturation to the density of the concrete specimens. As such, the saturation condition also affected the dynamic Poisson’s ratio of the concrete cylinders since the values were based on the P-wave and shear wave velocities.
3. The elastic moduli of the concrete specimen were affected differently by the saturation condition. The dynamic modulus of elasticity increased as the saturation also increased. Based on the standard *E_d,LR_*, MIX 1 exhibited the highest increase with 12.25% increase from OD to SSD condition, followed by Mix 2 with 10.25%, and then Mix 3 with 7.09%. The static modulus of elasticity also increased but not as much as the dynamic elastic modulus. The increase for Mixes 1 and 2 were about 1-2% while the elastic modulus for Mix 3 decreased by 3.9%. From this observation, it can be said that the effect of water saturation was more evident in the dynamic elastic modulus of concrete than its static elastic modulus.
4. The relationship between the static and dynamic elastic moduli was also investigated. It was shown that both the empirical equations (Equations (15) and (16)) from previous studies and the proposed equation from this research ($E_{d}=aE_{s}^{b})$ were effective in representing the relationship between the static and dynamic moduli of elasticity regardless of the saturation condition. The empirical equation proposed by Lydon and Balendran shows better agreement with the data from partial saturation conditions in this research. The proposed equation could be used as a reference in evaluating elastic moduli of concrete, which were under different saturation conditions.
5. The compressive strength of concrete from all mix proportions decreased as the saturation level increased. Among the three concrete mix proportions, MIX 3 established the highest reduction in compressive strength, followed by MIX 2 and then MIX 1. In this study, it could be interpreted that the lowest water-to-cement ratio would be more sensitive to the increased water saturation. However, it is suggested that further investigation on the effect of water saturation should be done involving the porosity and different material constituents of concrete materials.
6. The relationship between the stress wave velocities and the compressive strength was also investigated. The compressive strength decreased as the P-wave velocities increased. This observation was true for all the mix proportions used in this study. P-wave was greatly affected by the amount of water present in the concrete and other factors as shown in this study. Thus, estimation of compressive strength using P-wave velocity was not considered a proper method. In contrast, shear wave velocity was closely correlated with compressive strength of concrete, which was much less sensitive to water saturation level compared to compressive velocity.
7. This study was based on the laboratory investigations and might not fully represent the environmental factors affecting concrete in the field. This study only focused on water saturation. Saturation with different liquid may affect the behavior of concrete when the saturation level increases. Hence, it is also suggested that further investigations using other fluid that may affect concrete be investigated, such as chloride, among others.

## Figures and Tables

**Table 1:** Properties of the concrete cylinders used in this study.

	W/B (%)	S_V_/A_V_	Mixture Proportion (kg/m^3^)						
			W	C	S	G	SCMs		Chemical Admixture
							FA	SC	AE
MIX 1	51.52	0.490	170	99	884	931	33	198	1.98
MIX 2	35.43	0.495	170	110	858	923	37	220	2.57
MIX 3	25.60	0.456	160	312	725	876	63	250	6.88

Note W: water, B: binder, S_V_: volume of sand, A_V_: volume of aggregates, W: water, C: Portland cement type I, S: sand, G: gravel, SCMs: Supplementary cementitious materials; FA: fly ash type II, SC: Blast furnace slag cement type II, AE: high performance air-entraining agent.

**Table 2:** Chemical and physical composition of binders.

Type		Cement	Blast Furnace Slag Cement	Fly Ash
Fineness (cm^2^/g)		3.266	4.090	3.900
Density (g/cm^3^)		3.15	3.05	2.21
Chemical composition (%)	SiO_2_	20.68	26.32	50.72
	Al_2_O_3_	5.51	10.51	20.76
	Fe_2_O_3_	3.10	1.67	6.37
	CaO	62.28	55.39	1.82
	MgO	3.38	2.40	1.08
	SO_3_	2.56	2.80	0.62
	IOL (ignition loss)	1.42	0.91	1.34

**Table 3:** Basic physical properties of chemical admixture.

Type	Class	Color	Main Ingredient	Density [g/cm^3^]
Super plasticizer	Liquid	Lemon yellow	Polycarboxylate	1.04 ± 0.05

**Table 4:** Measured saturation levels of concrete cylinders in this study.

Saturation Degree		25%	50%	75%	100%
Mix 1	1	25.16	49.12	77.06	100.00
	2	24.97	51.36	73.03	97.47
	3	25.73	51.41	74.05	100.00
	4	24.70	49.93	75.90	97.37
	5	25.74	50.58	75.00	97.74
	Ave	25.26	50.48	75.01	98.52
Mix 2	1	26.35	50.97	76.12	99.69
	2	23.84	49.10	73.73	97.84
	3	26.14	48.80	73.17	100.00
	4	25.74	49.16	75.47	97.69
	5	26.39	50.09	74.09	100.00
	Ave	25.69	49.62	74.51	99.04
Mix 3	1	26.45	48.62	76.01	97.83
	2	24.43	50.38	75.92	100.00
	3	23.65	49.22	74.79	100.00
	4	26.25	51.98	74.16	98.60
	5	26.00	48.75	76.94	99.59
	Ave	25.36	49.79	75.57	99.20

**Table 5:** Regression analysis parameters of the relationship between static and dynamic elastic moduli across all mixes for each saturation condition.

Saturation Condition	a	b	R^2^
OD	0.4688	1.1885	0.93
25%	0.5111	1.1617	0.98
50%	0.379	1.2413	0.99
75%	0.4451	1.1957	0.98
SSD	0.4325	1.1798	0.98

## Data Availability

Data are contained within the article. But the data presented in this study are also available on request from the corresponding author.
